# A rare mitochondrial disorder: Leigh sydrome - a case report

**DOI:** 10.1186/1824-7288-36-62

**Published:** 2010-09-15

**Authors:** Dhananjay Y Shrikhande, Piyush Kalakoti, MM Aarif Syed, Kunal Ahya, Gurmeet Singh

**Affiliations:** 1 Department of Pediatrics, Rural Medical College, Loni, Maharashtra, India; 2 Rural Medical College, Loni, Maharashtra, India

## Abstract

Leigh syndrome is a rare progressive neurodegenerative, mitochondrial disorder of childhood with only a few cases documented from India. The clinical presentation of Leigh syndrome is highly variable. However, in most cases it presents as a progressive neurological disease with motor and intellectual developmental delay and signs and symptoms of brain stem and/or basal ganglia involvement. Raised lactate levels in blood and/or cerebrospinal fluid is noted. It is the neuroimaging, mainly the Magnetic Resonance Imaging showing characteristic symmetrical necrotic lesions in the basal ganglia and/or brain stem that leads to the diagnosis. Here, we report a case of 7 months old female child presenting to us with status epilepticus, delayed developmental milestones and regression of the achieved milestones suspected to be a case of neurodegenerative disorder, which on MRI was diagnosed as Leigh syndrome.

## Background

Leigh Syndrome [1], also termed as subacute necrotising encephalopathy is a rare, inherited progressive neurodegenerative disorder with characteristic pathological features usually presenting in infancy or early childhood. It was first reported in 1951 by Denis Leigh [2], a British neuropathologist, in a 7 month old infant that progressed rapidly and resulted in death over a 6-week period. Clinically, Leigh syndrome is characterized by psychomotor delay or regression, muscular hypotonia, brainstem signs (especially strabismus, nystagmus and swallowing difficulties), ataxia, pyramidal signs, respiratory insufficiency, lactate acidemia and acute deterioration following common infections. In most cases, dysfunction of the respiratory chain enzymes is responsible for the disease. It may be due to defects in genes for the pyruvate dehydrogenase complex, cytochrome-c oxidase, ATP synthase subunit 6, or subunits of mitochondrial complex I. Patterns of inheritance include X-linked recessive, autosomal recessive, and mitochondrial [3]. However the genetic cause of a number of cases of Leigh syndrome remains unknown, despite the presence of a specific biochemical defect in many of them. Despite its considerable clinical, genetic and biochemistry heterogeneity, the basic neuropathological features in children affected are almost identical; which are focal, bilateral, and symmetric necrotic lesions associated with demyelination, vascular proliferation and gliosis in the brainstem, diencephalon, basal ganglia, and cerebellum [4]. It is possible to come to a diagnosis of probable SNE during life on the basis of clinical signs and symptoms, mode of inheritance, metabolic abnormalities, and neuroimaging findings [5]. We report a rare case which presented clinically as a neurodegenerative disorder and diagnosed as Leigh syndrome on MRI.

## Case Presentation

A 7 month old female child, 2^nd ^product of second degree consanguineous marriage, with an uneventful perinatal history presented to our hospital with status epilepticus, delayed developmental milestones and regression of the achieved milestones. On initial examination, she was unconscious (Glassgow Coma Scale-5) and afebrile. Initial management aimed at controlling the seizures with Diazepam (i.v., 0.3 mg/kg stat) and Phenytoin (i.v., 20 mg/kg stat followed by 5 mg/kg per 12 hourly). Following control of seizures the child went into decerebrate posturing. The raised intracranial tension was treated with Mannitol (i.v., 5 mg stat). Her pulse was 154 beats per minute, respiratory rate 36 cycles per minute and blood pressure 84/46 mm Hg. Her weight was 5 kg and height 62 cms. CNS examination showed increased tone in the lower limbs. Deep tendon reflexes were exaggerated with bilateral Babinski sign. Pupils were dilated and sluggishly reacting to light. Fundus examination and visual evoked potentials were normal. After an hour of admission, she became apnoeic and was put on ventilator. The above clinical findings were highly suggestive of a neurodegenerative disorder and the patient was further investigated.

**Figure 1:**
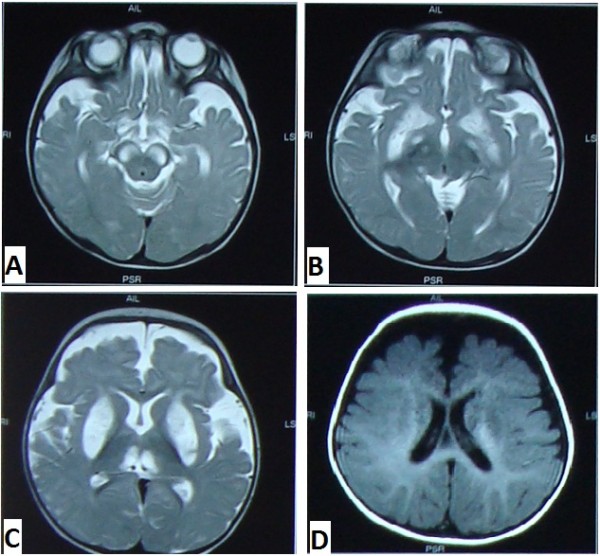
**MRI Findings of Leigh Syndrome.** A & B: T2W image showing bilateral symmetrical abnormal signal intensities, seen in cerebral peduncles, dorsal medulla and peri aqueductal grey matter. C: T2W image showing bilateral symmetrical abnormal signal intensities, seen involving basal ganglia and thalami. D: T2W image showing prominent extracerebral CSF spaces in fronto-temporo-parietal region on both sides depicting signs of frontal atrophy

Routine haemogram revealed haemoglobin 8.8 gm%, packed cell volume 28.6%, total leucocyte count 26,800 cells per mm^3 ^with marked neutrophilia (85%) and lymphocyte count 10%. Cerebrospinal fluid examination showed 4 cells, all lymphocytes and normal sugar and protein levels. CSF lactate was significantly raised (8.8 mmol/L). Gram and ZN staining of the CSF showed no organism and pus cells. Serum lactate (6.8 mmol/L) and creatinine kinase (320 U/L) levels were abnormally raised. Liver function test showed mild derangement with AST- 54 IU/L, ALT- 49 IU/L and ALP- 109 IU/L. Renal function test was within normal limits. Arterial blood gas analysis indicated metabolic acidosis. Blood and urine cultures were negative. Magnetic Resonance Imaging was done which showed bilateral, symmetrical abnormal lesions in the basal ganglia, thalami, cerebral peduncles, dorsal medulla and peri aqueductal grey matter which were hyperintense in T2W, FLAIR and DW images (Figure 1A, B &1C). There were prominent extracerebral CSF spaces in the fronto-temporo-parietal region on both the sides and showed the similar signal characteristics (Figure 1D). Frontal atrophy with myelination normal for age was noticed. Above radiological findings on MRI established the clinical diagnosis of a neurodegenerative disorder as Leigh syndrome.

Supportive therapy for the suspected mitochondrial disorder was begun with intravenous Thiamine infusions, Carnitine, alkali supplementation and oral coenzyme Q_10_(Ubiquinone). Over the next 2 days, she improved clinically and was extubated. However, the next few hours were critical, her conditions deteriorating and she went into respiratory arrest requiring reintubation. She went into coma with GCS 4. Oculocephalic reflex became absent. The patient eventually died 10 days after admission.

## Discussion

Leigh's disease or SNE is a rare progressive neurological disorder of the childhood. The estimated prevalence of Leigh Syndrome was 2.05 cases per 1, 00,000 [6]. The preschool incidence of Leigh syndrome was 1 out of 32000 [7]. Age of onset of symptoms is usually less than 2 years (infantile form), but others may present in childhood (juvenile form) and unusually in adulthood. It presents early in life with psychomotor regression, abnormal muscle tone, weakness, dystonia, brainstem and cerebellar dysfunction (ataxia), visual loss, missed milestones or regression of the achieved milestones, tachypnea, and seizures [2,8,9]. Affected children usually become symptomatic within the first year of life with feeding difficulties, vomiting and failure to thrive [1]. Death usually occurs within a few years after onset of symptoms, typically from progressive respiratory failure [10,11]. Laboratory analysis shows metabolic acidosis with elevated blood, CSF lactate, and pyruvate concentrations [8]. It is usually inherited in an autosomal recessive fashion. The underlying defect can be at any of the sites in the enzyme pathway for respiratory metabolism. Associated mitochondrial enzyme deficiencies are those of pyruvate carboxylase, pyruvate dehydrogenase, cytochrome C oxidase, and Complex 1 (NAD-Coenzyme Q Reductase) [8,10]. The diagnostic criteria are: (1) Progressive neurological disease with motor and intellectual developmental delay; (2) Signs and symptoms of brainstem and/or basal ganglia disease; (3) Raised lactate levels in blood and/or cerebrospinal fluid; (4) Characteristic symmetric necrotic lesions in the basal ganglia and/or brainstem [4].

Neuroimaging [8,12-15] plays an important role in diagnosis of patients with Leigh syndrome. The most characteristic neuro-radiological findings are bilateral, symmetric focal hyperintensities in the basal ganglia, thalamus, substantia nigra, and brainstem nuclei at various levels on T2-weighted MRI. These high T2 signals on MRI reflect the spongiform changes and vacuolation in the affected brain structures [16-18]. In the basal ganglia, the putamen is particularly involved. In one series, 100% of the patients with proven SNE had putaminal involvement [8]. Ghosh and Pradhan [13] reported two children with Leigh syndrome suspected clinically and confirmed by MRI in 1996. Low attenuation in the putamina on CT is considered to be characteristic of the disease [11,19]. In India, Bhavsar VM, Kumta NB [12] described the role of CT scan of the brain in the diagnosis of Leigh syndrome in 1991. In 2004, Mannan and Sharma et al [20] reported autopsy proven Leigh syndrome in a 15-month-old girl admitted with cough and hyperventilation. In 2005, Hombal and Narvekar [14] reported Leigh syndrome in a 3-year-old child with regression of milestones and involuntary movements. The diagnosis in their case was based on neuroimaging.

Specific therapy for mitochondrial disorders in children is not available. The results and prognosis are variable. The aim of symptomatic treatment is to improve the ATP production and to lower the lactate levels. Thiamine, a cofactor of pyruvate dehydrogenase complex has been reported to improve the neurological status in some patients [21]. Marked improvement was observed with riboflavin, which nearly normalized the adenosine triphosphate production [22,23]. Rapid clinical and biochemical improvement was observed in patients with acute central respiratory failure with the use of intravenous soya bean oil (ketogenic emulsion) [24]. Ketogenic diet has been found to improve the outcome in those with a deficiency of pyruvate dehydrogenase [25]. Coenzyme Q and Carnitine [26] have also been found to be effective. Leung TF, Hui J et al described significant relief of dystonia with intramuscular botulinum toxin [27]. Nucleus transplantation into enucleated oocyte is emerging as a new option for prevention of mitochondrial disorders [28].

This child presented to us with seizures, regression of developmental milestones and acute exacerbation caused by a trivial respiratory illness. These symptoms pointed towards a neurodegenerative disorder. Examination revealed delayed development, hypertonia and disorientation, all of which are recognized features of Leigh Syndrome [1]. CSF lactate was markedly elevated, but arterial lactate was normal. Though Leigh Syndrome is conventionally associated with elevated serum lactate, earlier studies have shown that serum lactate can be well within normal limits in spite of definite neuro-radiological features and spectroscopic evidence of elevated brain lactate [29]. The imaging findings suggested a progressive neurodegenerative disorder with the possibility of a mitochondrial encephalopathy. This is consistent with the neuro-radiological findings in previous reports of Leigh Syndrome [8,12-15]. Enzymology, histology and functional fibroblast ATP synthesis rate, molecular studies were not performed due to the paucity of facilities and financial constraints.

## Conclusion

The diagnosis of Leigh's disease should be considered in appropriate clinical and laboratory settings whenever symmetrical hypodensities are encountered in the putamina and midbrain on CT and further investigated with MRI. Our experience suggested that bilateral symmetric T2 prolongation involving multiple brainstem nuclei/structures associated with basal ganglia abnormalities in a child with neurological problems should prompt the clinician to consider Leigh syndrome and conduct further investigations such as measurement of blood and/or CSF lactate, and respiratory chain enzymes activities. Neuro-sradiological discriminative observation is very useful in guiding the clinicians for the most appropriate enzymatic and genetic study in their patients. Mitochondrial disease cannot be cured completely. Efforts for prevention and prenatal diagnosis are still in the nascent stage. With appropriate investigations, accurate diagnosis and prompt institution of adequate supportive therapy, symptomatic amelioration can be achieved, thereby adding life to the limited years of survival of these children. Further research aimed at prenatal identification of the responsible mutations and prevention of the disease is warranted.

## Consent

Written informed consent was obtained from the father of the patient for publication of this case report and any accompanying images. A copy of the written consent is available for review by the editor-in-chief of this journal.

## List of abbreviations

MRI: Magnetic Resonance Imaging; SNE: Subacute necrotising encephalopathy; DNA: Deoxyribonucleic acid; GCS: Glassgow Coma Scale; i.v.: intravenous; CSF: Cerebrospinal fluid; AST: Aspartate transaminase; ALT: Alanine transaminase; ALP: Alkaline phosphatase; NAD: Nicotinamide adenine dinucleotide; CT: Computed Tomography; ATP: Adenosine triphosphate.

## Competing interests

The authors declare that they have no competing interests.

## Authors' contributions

DYS, PK, MMAS participated in the clinical diagnosis, sequence alignment, drafting the manuscript and made useful contribution in the revision of the literature. KA and GS participated in writing discussion. All authors read and approved the final manuscript.
